# Supplementary material for: Evaluation of Scab and Mildew Resistance in the Gene Bank Collection of Apples in Dresden-Pillnitz
Source: Plants (Basel). 2021 Jun 16;10(6):1227. doi: 10.3390/plants10061227 (PMC8234245; doi:10.3390/plants10061227)
Supplement: Supplementary file 1 [file plants-10-01227-s001.zip › plants-1238429-supplementary.pdf]

Table S1: Assessment of susceptibility to scab (leaf and fruit) and mildew of the apple cultivars in the JKI collection in 2012 and 2013. A nine-step scoring scale was used: 1 (no symptoms) and 9 (very heavy infection). Data for each cultivar (2 trees) with the heaviest infection were considered.

Cultivars	Acc. No.	Scab leaf 2012	Scab leaf 2013	Scab fruit 2012	Scab fruit 2013	Mildew leaf 2012	Mildew leaf 2013	Maximum score 3*
Äckerle-Apfel	APF1501	2	5	1	3	3	3	
Adams Parmäne	APF0002	1	4	1	1	1	3	
Adersleber Kalvill	APF0003	3	5	1	7	5	4	
Ahra	APF0749	1	1	1	3	3	3	x
Ahrina	APF0004	2	5	1	9	5	3	
Ahrista	APF0748	2	1	1	1	3	3	x
Aiwanija	APF1304	6	7	5	9	6	6	
Akane	APF0005	1	1	1	1	7	3	
Akerö	APF0287	2	5	1	5	7	6	
Alantapfel	APF1193	3	6	1	7	6	7	
Alkmene	APF0009	1	4	1	5	3	3	
Allendorfer Rosenapfel	APF1553	2	5	1	2	3	3	
Allington Pepping	APF0012	1	1	1	5	5	6	
Altländer Jakobsapfel	APF1195	3	6	1	3	3	6	
Altländer Pfannkuchenapfel	APF0015	1	2	1	3	2	2	x
Altländer Rosenapfel	APF0016	1	1	1	1	2	1	x
Altmärker Brautapfel	APF1383	2	n.d.	1	n.d.	2	n.d.	
Anacuta	APF0020	1	5	1	9	3	4	
Ananasrenette	APF0021	2	4	2	8	4	4	
Aneta	APF1113	1	1	1	2	3	2	x
Angelner Borsdorfer	APF1384	1	n.d.	1	n.d.	3	n.d.	
Angelner Herrenapfel	APF1305	1	4	1	5	4	4	
Angold	APF0902	1	1	1	1	5	4	
Anhalter	APF1197	3	7	1	5	4	1	
Annie Elizabeth	APF1306	2	7	1	7	7	7	
Antonovka	APF0022	1	1	1	1	4	4	
Antonovka Kamenička	APF0024	1	1	1	1	2	3	x
Antonovka Polutorafuntovaâ	APF0023	1	1	1	3	2	2	x
Apfel aus Grignon	APF1502	1	6	1	1	4	5	
Apfel aus Halder	APF0266	2	5	1	1	5	4	
Apfel aus Hawthornden	APF1337	2	2	1	4	2	4	
Apfel aus Lunow	APF0938	1	2	1	3	3	3	x
Api Etoile	APF1308	2	6	1	5	4	5	
Api Noir	APF1309	3	7	n.d.	9	3	5	
Api Rose	APF1198	3	6	2	3	5	6	

Cultivars	Acc. No.	Scab leaf		Scab fruit		Mildew leaf		Maximum score 3*
		2012	2013	2012	2013	2012	2013	
Apollo	APF0026	1	4	1	3	3	3	
Ariwa	APF1041	1	1	1	1	2	1	x
Arlet	APF0028	4	6	2	8	4	5	
Ashmeads Kernel	APF0936	2	4	1	1	1	2	
Astillisch	APF0032	2	6	1	5	3	4	
Astramel	APF0033	3	7	1	8	3	3	
Auralia	APF0035	3	3	1	5	4	4	
Ausbacher Roter	APF0940	2	5	1	5	3	2	
Backapfel	APF1200	3	7	1	4	3	4	
Baldwin	APF0038	4	5	1	2	6	5	
Bancroft	APF0040	3	5	2	5	5	5	
Bänziger	APF1310	1	4	1	5	3	3	
Batullenapfel	APF1385	2	n.d.	1	n.d.	3	n.d.	
Baujade	APF0878	5	1	1	3	3	4	
Baumanns Renette	APF0994	3	5	1	3	1	2	
Bayhs Mostapfel	APF1386	4	n.d.	1	n.d.	4	n.d.	
Beerbacher Taffetapfel	APF1201	1	6	n.d.	3	3	3	
Bellefleur Kitajka	APF0044	3	1	2	4	7	6	
Bellefleur Krasnij	APF0043	2	3	1	3	3	3	x
Benoni	APF1311	1	7	1	8	4	6	
Berkersheimer Roter	APF1202	3	5	n.d.	2	6	4	
Berlon	APF0047	3	4	1	3	5	4	
Berner Rosenapfel	APF0048	1	4	1	3	3	3	
Bessemânka Mičurinskaâ	APF0050	2	1	1	1	3	4	
Betzinger Grünapfel	APF1504	2	5	1	7	1	1	
Biesterfelder Renette	APF0051	1	4	1	n.d.	4	3	
Birnförmiger Apfel	APF1388	2	n.d.	1	n.d.	2	n.d.	
Bischofshut	APF0052	2	3	1	n.d.	3	4	
Bischofsmütze nach Zorn	APF1312	1	3	3	1	2	6	
Bismarckapfel	APF0053	3	5	1	5	4	6	
Bittenfelder	APF0054	1	4	1	3	3	1	
Blauacher Wädenswil	APF0594	4	7	1	7	4	5	
Blumberger Langstiel	APF1313	1	4	1	7	3	3	
Böblinger Straßenapfel	APF1505	1	5	1	3	1	1	
Bockenhusen	APF1205	2	3	1	1	2	1	x
Böhmischer Roter Jungfernapfel	APF1194	3	3	1	5	5	5	
Boikenapfel	APF0061	1	5	1	7	3	6	
Borsdorfer Kitajka	APF0064	1	5	1	8	3	5	
Börtlinger Weinapfel	APF0065	2	1	1	1	2	2	x
Boskoop	APF0066	1	4	1	1	2	3	

Cultivars	Acc. No.	Scab leaf 2012	Scab leaf 2013	Scab fruit 2012	Scab fruit 2013	Mildew leaf 2012	Mildew leaf 2013	Maximum score 3*
Braeburn	APF0073	4	8	1	9	7	8	
Bramleys Seedling	APF0074	2	3	1	1	2	2	x
Bratwurstapfel	APF1389	2	n.d.	1	n.d.	3	n.d.	
Bratzelapfel	APF1506	3	3	1	5	2	2	
Bremer Doodapfel	APF1206	7	7	5	7	6	4	
Brettacher	APF0077	2	1	3	2	3	3	x
Brownless Russet	APF1314	2	6	1	1	3	3	
Burchards Netzrenette	APF1393	1	n.d.	1	n.d.	2	n.d.	
Burgstädter Renette	APF1207	2	6	1	5	3	4	
Carola	APF0099	2	5	1	5	5	6	
Caroline Hopkins	APF1315	6	7	1	7	2	3	
Carpentin	APF1208	3	6	1	1	3	3	
Celeste	APF1134	5	6	5	9	1	4	
Cellini	APF0100	2	6	1	8	2	4	
Champagner Renette	APF0101	1	6	1	5	1	4	
Charlamowsky	APF0103	2	4	1	5	4	6	
Charles Ross	APF0104	2	3	1	3	2	4	
Charlotte	APF0105	4	7	1	9	4	6	
Clivia	APF0111	3	5	2	8	8	6	
Cloden	APF0234	3	7	3	9	5	5	
Cludius Herbstapfel	APF1209	1	5	3	9	5	6	
Coulons Renette	APF0118	1	3	1	1	3	4	
Cox Orange	APF0120	3	1	1	1	6	7	
Cox Pomona	APF0121	2	4	1	7	3	4	
Crawley Beauty	APF0123	3	3	3	5	3	3	
Creo	APF0996	3	6	2	3	3	3	
Crimson Beauty	APF0124	2	5	1	3	3	4	
Crimson Crisp	APF0923	2	1	1	1	6	5	
Cripps Pink	APF1044	4	8	1	9	4	4	
Cripps Red	APF1045	7	7	4	9	4	4	
Dalili	APF1132	6	4	9	9	3	4	
Dalinbel	APF1053	4	3	1	3	7	6	
Dalinred	APF1054	2	1	1	2	4	4	
Damason Renette	APF0995	2	7	1	1	3	4	
Danziger Kantapfel	APF0598	2	3	1	1	4	3	
Dayton	APF0907	2	4	1	1	3	4	
Dean's Küchenapfel	APF1316	2	2	1	7	6	7	
Delcorf	APF0133	4	5	7	7	3	5	
Delgollune	APF0132	3	7	3	8	3	4	
Delicious	APF0135	4	6	4	5	5	4	

Cultivars	Acc. No.	Scab leaf 2012	Scab leaf 2013	Scab fruit 2012	Scab fruit 2013	Mildew leaf 2012	Mildew leaf 2013	Maximum score 3*
Delorina	APF1112	3	3	1	1	6	7	
Demokrat	APF0148	3	5	2	9	2	4	
Der Leckerbissen	APF1508	2	n.d.	1	n.d.	4	4	
Diana	APF0151	2	3	1	7	5	4	
Dietzer Wintergoldrenette	APF1210	1	2	1	3	3	1	x
Discovery	APF0153	1	1	1	1	3	3	x
Doberaner Renette	APF1318	3	6	1	n.d.	3	1	
Doppelter Härtling	APF1510	2	4	1	5	4	5	
Doppelter Hausapfel	APF1511	3	n.d.	1	n.d.	3	4	
Doppelter Prinzenapfel	APF1319	1	4	1	n.d.	3	3	
Dorheimer Streifling	APF1212	1	1	1	1	5	3	
Drakenstein	APF0886	5	5	3	9	5	6	
Dukat	APF0158	8	7	7	9	3	6	
Dülmener Rosenapfel	APF0159	2	1	1	3	2	3	x
Early McIntosh	APF0161	2	3	2	n.d.	1	5	
Ecolette	APF0998	1	1	1	2	3	2	x
Edelborsdorfer	APF1131	1	5	1	3	1	3	
Edelroter	APF1213	3	6	1	7	3	4	
Edler aus Leipzig	APF0200	3	7	1	n.d.	3	4	
Edler Rosenstreifling	APF1215	2	7	n.d.	5	6	7	
Edward VII	APF1320	2	4	3	5	1	3	
Egremont Russet	APF1321	3	5	1	1	4	6	
Elan	APF0170	5	5	1	9	3	7	
Elektra	APF0172	4	4	1	7	6	7	
Elise Rathke	APF0897	2	4	2	n.d.	3	4	
Elstar	APF0176	5	5	2	1	6	8	
Empire	APF0178	4	7	1	9	3	5	
Engelsberger	APF0179	1	5	1	5	2	4	
Engelshofer	APF0180	1	1	1	1	2	3	x
Englische Spitalrenette	APF1216	3	4	1	3	4	3	
Englischer Prinz	APF0181	1	1	1	1	3	4	
Enterprise	APF0914	1	3	1	1	3	4	
Erbachhofer	APF0894	1	1	1	3	2	1	x
Ernst Bosch	APF0410	1	1	1	n.d.	3	5	
Erwin Baur	APF 0185	4	8	2	1	1	4	
Erwin Junge	APF0186	3	6	3	9	7	7	
Esopus Spitzenburg	APF1217	4	7	3	n.d.	7	6	
Eva's Apfel	APF1402	5	n.d.	1	n.d.	3	n.d.	
Extertaler	APF1218	2	3	1	1	3	3	x

Cultivars	Acc. No.	Scab leaf 2012	Scab leaf 2013	Scab fruit 2012	Scab fruit 2013	Mildew leaf 2012	Mildew leaf 2013	Maximum score 3*
Falstaff	APF0188	5	5	3	9	6	7	
Fameuse	APF1403	3	n.d.	1	n.d.	2	n.d.	
Fantazja	APF0189	3	8	3	9	5	7	
Fettapfel	APF1324	1	3	2	3	2	3	x
Feuerroter Taubenapfel	APF0291	2	n.d.	1	7	4	5	
Fießers Erstling	APF0191	2	2	3	1	4	4	
Fiesta	APF0192	5	6	1	8	6	7	
Filippa	APF1325	1	3	1	3	3	3	x
Finkenwerder Prinzenapfel	APF0194	2	2	1	1	4	4	
Florina	APF1456	5	6	1	1	3	1	
Fraas Sommerkalvill	APF1327	1	3	1	6	5	6	
Franksenapfel	APF0948	1	1	1	1	5	7	
Französische Goldrenette	APF1328	2	7	1	3	5	7	
Französischer Tiroler	APF0196	2	3	2	9	4	6	
Frau Margarethe von Stosch	APF1329	5	7	4	n.d.	5	7	
Freiherr von Berlepsch	APF0046	1	5	1	5	4	5	
Freiherr von Hausen	APF1182	2	7	1	9	3	4	
Freyberg	APF0198	6	4	1	1	5	9	
Friedberger Bohnapfel	APF1408	1	n.d.	1	n.d.	2	n.d.	
Friesenapfel	APF1512	2	3	1	1	3	3	x
Früher Viktoria	APF0202	2	1	1	1	2	3	x
Fuji	APF0204	6	7	5	9	3	4	
Fukunishiki	APF0205	7	7	1	5	4	4	
Fürst Blücher	APF0207	1	1	1	3	3	4	
Gacksapfel	APF1221	1	6	1	5	3	3	
Gaesdonker Renette	APF1513	2	1	1	1	1	1	x
Gala	APF0208	5	5	3	9	5	5	
Galloway Pepping	APF0210	2	6	1	1	5	4	
Gartenmeister Simon	APF1409	2	n.d.	1	n.d.	3	n.d.	
Gascoynes Scharlachroter	APF0212	2	7	1	1	2	3	
Geflammter Kardinal	APF1330	2	1	1	1	3	3	x
Geheimrat Breuhahn	APF0078	2	7	1	9	5	5	
Geheimrat Dr. Oldenburg	APF0486	5	7	3	9	2	4	
Geheimrat Wesener	APF1410	3	n.d.	1	n.d.	3	n.d.	
Gehrers Rambur	APF1120	2	3	1	3	3	3	x
Gelbe Sächsische Renette	APF0225	2	6	1	3	4	5	
Gelbe Schleswiger Renette	APF1331	1	1	1	3	3	2	x
Gelber Bellefleur	APF0227	2	6	1	n.d.	4	4	
Gelber Edelapfel	APF0226	2	5	1	n.d.	3	4	
Gelber Münsterländer Borsdorfer	APF0943	1	4	1	3	5	4	

Cultivars	Acc. No.	Scab leaf 2012	Scab leaf 2013	Scab fruit 2012	Scab fruit 2013	Mildew leaf 2012	Mildew leaf 2013	Maximum score 3*
Gelber Osterapfel	APF1411	2	n.d.	1	n.d.	5	n.d.	
Gelber Richard	APF0249	2	7	1	7	5	7	
Generos	APF1039	1	1	1	1	6	6	
Georg Cave	APF1412	5	n.d.	1	n.d.	4	n.d.	
Gerlinde	APF0992	2	5	1	3	3	4	
Gestreifter Matapfel	APF1203	3	4	1	2	7	3	
Gewürzluiken	APF0939	1	4	1	2	2	3	
Gilliflower	APF1395	1	n.d.	1	n.d.	3	n.d.	
Glogierowka	APF0236	1	5	1	4	4	3	
Gloria Mundi	APF1046	2	5	1	n.d.	2	4	
Gloster	APF0238	3	7	1	9	2	6	
Gniedelsteiner	APF1225	4	5	1	5	4	4	
Gochsheimer	APF1514	1	2	1	1	1	1	x
Göhrings Renette	APF1563	1	3	1	1	2	1	x
Golden Delicious	APF0239	6	7	5	9	5	7	
Golden Delicious Belgolden	APF0042	5	7	8	n.d.	5	8	
Golden Delicious Bovey 85	APF0215	6	7	3	9	5	7	
Golden Delicious Doud	APF0137	7	8	3	n.d.	5	8	
Golden Delicious Smoothee	APF0640	5	7	3	9	5	7	
Golden Resistant	APF0241	1	2	1	3	2	3	x
Goldparmäne	APF0724	2	5	1	3	3	5	
Goldrenette aus Blenheim	APF1079	3	6	1	3	2	3	
Goldrenette Römischer Kikker	APF1332	3	5	1	9	7	7	
GoldRush	APF0922	1	2	1	1	6	7	
Goldstar	APF1114	1	4	1	1	3	4	
Graf Ezzo	APF0247	5	6	2	6	2	5	
Grahams Jubiläumsapfel	APF0648	1	4	1	4	1	4	
Granny Smith	APF0250	3	8	2	8	5	8	
Grasblümchen	APF1414	2	n.d.	1	n.d.	4	n.d.	
Graue Herbstrenette	APF1228	4	7	1	1	3	3	
Gravensteiner	APF0253	2	7	1	5	3	5	
Greensleeves	APF0255	3	6	1	4	4	4	
Grenadier	APF0256	2	1	1	1	1	3	x
Gretapfel	APF1333	1	1	1	1	4	3	
Grimes Golden	APF0258	4	6	3	9	5	6	
Groninger Krone	APF0946	1	3	1	3	3	6	
Großer Api	APF1230	4	7	1	3	1	2	
Großherzog Friedrich von Baden	APF1334	2	4	1	7	5	5	
Grünapfel	APF0018	1	4	1	3	2	3	
Grüner Fürstenapfel	APF1232	1	3	1	3	4	3	

Cultivars	Acc. No.	Scab leaf 2012	Scab leaf 2013	Scab fruit 2012	Scab fruit 2013	Mildew leaf 2012	Mildew leaf 2013	Maximum score 3*
Grüner Stettiner	APF1322	2	4	1	n.d.	4	3	
Grüner Wilhelm	APF1517	2	3	1	3	5	3	
Grünling von Rhode Island	APF0574	2	6	1	3	6	6	
Gubener Waraschke	APF0261	1	3	1	1	3	1	x
Gustavs Dauerapfel	APF1335	1	2	1	2	3	2	x
Hadelner Sommerprinz	APF1336	1	2	1	1	3	2	x
Halberstädter Jungfernapfel	APF1338	3	7	1	8	3	4	
Hansa	APF1419	2	7	1	7	3	3	
Harberts Renette	APF0268	3	7	1	9	4	7	
Hartapfel	APF1233	3	5	1	7	2	4	
Hauschildapfel	APF1421	2	7	1	7	5	5	
Hausmütterchen	APF1422	2	6	1	5	3	4	
Hauxapfel	APF0270	2	7	1	3	1	5	
Havelgold	APF0271	4	7	2	9	6	7	
Heimeldinger	APF1235	3	4	1	2	3	5	
Heinemanns Schlotterapfel	APF1181	1	1	1	1	3	5	
Helios	APF0272	4	5	3	n.d.	3	3	
Herbststreifling	APF1234	2	6	1	3	6	8	
Herma	APF0274	6	8	n.d.	9	5	8	
Hermann	APF1423	2	4	1	5	3	4	
Herzog von Cumberland	APF1424	1	3	1	4	3	2	
Herzogin Olga	APF1425	4	4	1	n.d.	3	5	
Heslacher Gereutapfel	APF1237	1	3	n.d.	2	3	3	
Hesselmanns Schlotterapfel	APF1339	1	4	1	1	3	4	
Heuchelheimer Schneeapfel	APF0276	2	3	1	3	3	4	
Hibernal	APF0277	1	1	1	1	2	3	x
Hibernal 4n	APF0278	1	1	n.d.	3	2	2	
Hilde	APF0891	1	1	1	3	2	4	
Himbacher Grüner	APF1238	3	6	2	7	4	4	
Himbeerapfel aus Holovousy	APF0279	1	3	5	5	4	5	
Hochzeitsapfel	APF1240	2	2	1	1	2	3	x
Holiday	APF0280	5	8	5	9	5	7	
Holländer Prinz	APF1241	1	5	1	3	3	3	
Holsteiner Cox	APF1340	3	6	1	n.d.	5	6	
Honeygold	APF0282	5	7	3	9	4	6	
Honigmilchapfel	APF1428	3	5	1	5	3	4	
Horei	APF0283	5	8	4	9	5	8	
Horneburger Pfannkuchenapfel	APF0284	1	6	1	5	3	4	
Horrenberger Renette	APF1520	3	5	1	7	3	2	
Hossfelds Gulderling	APF0262	1	3	1	5	4	4	

Cultivars	Acc. No.	Scab leaf 2012	Scab leaf 2013	Scab fruit 2012	Scab fruit 2013	Mildew leaf 2012	Mildew leaf 2013	Maximum score 3*
Howgate Wonder	APF0285	2	7	1	9	2	3	
Idagold	APF0288	2	8	1	8	5	7	
Idared	APF0290	5	7	n.d.	9	7	9	
Iduna	APF1042	4	7	1	9	3	5	
Ilga	APF0292	1	1	1	5	3	3	
Ingol	APF0294	1	4	1	n.d.	4	4	
Ingrid Marie	APF0295	1	2	1	n.d.	4	4	
Iversenapfel	APF1429	3	6	1	5	4	6	
Ivette	APF0296	4	7	3	5	5	7	
Jakob Fischer	APF0297	1	2	1	n.d.	1	2	
Jakob Lebel	APF0298	1	5	3	6	2	3	
Jamba	APF0299	3	6	1	5	3	4	
James Grieve	APF0300	4	6	3	6	3	7	
Jerseymac	APF0306	5	7	5	9	4	7	
Jester	APF0307	3	5	4	5	3	5	
Jeverländer Süßapfel	APF1243	1	1	1	1	1	1	x
Johannes Böttner	APF0308	3	6	3	7	3	7	
Johannes Hannes	APF1522	2	3	n.d.	n.d.	5	5	
Jonadel	APF0312	1	4	1	1	2	4	
Jonagold	APF0315	5	8	3	8	7	8	
Jonagored	APF0317	7	8	6	8	7	8	
Jonagram	APF0318	9	9	5	9	3	7	
Jonamac	APF0321	7	8	5	9	3	5	
Jonathan	APF0327	4	7	3	9	5	7	
Jongrimes	APF0330	1	4	1	5	3	4	
Josef Musch	APF1342	3	7	1	3	3	3	
Joyce	APF1025	3	7	1	3	2	3	
Ju Tsin	APF0333	2	7	1	7	6	5	
Juliane	APF0339	1	3	1	n.d.	2	1	
Juno	APF0342	5	7	4	7	5	7	
Jupiter	APF0343	4	4	1	n.d.	6	5	
Kaiser Alexander	APF0926	2	5	1	5	2	4	
Kaiser Wilhelm	APF0346	2	3	1	n.d.	3	4	
Kanadarenette	APF0348	3	5	1	1	3	3	
Kandil Sinap	APF1344	1	5	1	n.d.	4	5	
Kardinal Bea	APF0349	1	3	1	3	1	2	x
Karin Schneider	APF0350	1	2	1	3	4	4	
Karmeliter Renette	APF0352	1	7	1	5	5	5	
Karmijn de Sonnaville	APF0354	3	5	1	n.d.	5	6	
Karthäuserapfel	APF1433	2	7	1	7	2	3	

Cultivars	Acc. No.	Scab leaf 2012	Scab leaf 2013	Scab fruit 2012	Scab fruit 2013	Mildew leaf 2012	Mildew leaf 2013	Maximum score 3*
Käsapfel	APF1244	1	5	1	1	3	4	
Kaschaker	APF0355	4	5	3	6	1	4	
Kasseler Renette	APF0356	2	7	n.d.	7	3	4	
Kendall	APF0358	5	7	1	9	4	7	
Keswick's Küchenapfel	APF1245	1	3	2	2	3	3	x
Kidd's Orange Red	APF0361	7	5	1	n.d.	5	6	
Kirschweinling	APF0364	1	5	1	4	2	3	
Kitajka Solotaâ Rannââ	APF0365	1	5	1	7	3	5	
Klarapfel	APF0367	1	5	1	1	4	5	
Kleiner Fleiner	APF1434	1	6	1	3	2	4	
Kleiner Langstiel	APF1246	1	5	1	4	3	6	
Kloppenheimer Streifling	APF1247	1	6	1	1	3	3	
Knäckerla	APF1523	1	1	n.d.	1	4	6	
Königin Sophienapfel	APF1345	2	5	1	7	3	3	
Königinapfel	APF0368	3	5	1	3	1	4	
Königlicher Kurzstiel	APF0369	2	5	1	n.d.	3	5	
Königsapfel von Jersey	APF1248	1	1	n.d.	1	3	4	
Korbacher Edelrenette	APF1249	1	1	1	3	4	3	
Korei	APF0371	5	8	4	9	5	7	
Kriemhild	APF0257	3	5	1	4	3	4	
Kronprinz Rudolf	APF1436	4	7	3	3	3	4	
Krügers Dickstiel	APF0375	1	4	1	1	2	2	
Krumstedter Paradies	APF1252	3	6	1	1	6	7	
Kumpfenapfel	APF1524	1	2	1	3	2	6	
Kurzcox	APF0377	3	4	1	3	4	5	
Lady Apple	APF1253	4	8	1	9	3	7	
Lady Williams	APF0930	5	9	1	9	4	5	
Landsberger Renette	APF0378	5	7	5	9	3	4	
Lanes Prinz Albert	APF0379	4	6	1	5	5	6	
Langer Grüner Gulderling	APF1255	3	6	1	n.d.	5	4	
Langtons Sondergleichen	APF1257	1	5	1	7	3	3	
Large Transparent	APF0381	2	7	1	n.d.	6	7	
Lausitzer Nelkenapfel	APF0383	2	6	1	6	3	4	
Lautertaler Waldapfel	APF1438	2	3	1	5	2	3	
Laxtons Fortune	APF0388	1	2	1	1	2	2	x
Laxtons Superb	APF0392	4	6	1	n.d.	4	4	
Laxtons Triumph	APF0393	1	4	1	n.d.	4	4	
Leistadter Rotapfel	APF1439	2	5	5	5	1	2	
Leupoldsdorfer Süßapfel	APF0394	1	4	1	1	5	6	
Linda	APF0396	2	5	n.d.	7	3	5	

Cultivars	Acc. No.	Scab leaf 2012	Scab leaf 2013	Scab fruit 2012	Scab fruit 2013	Mildew leaf 2012	Mildew leaf 2013	Maximum score 3*
Linsenhofer Sämling	APF1347	1	2	1	1	2	2	x
Lippoldsberger Tiefblüte	APF1487	1	5	1	5	2	3	
Lired	APF0301	4	7	3	7	3	7	
Lobo	APF0397	4	7	4	9	4	5	
Lohrer Rambur	APF0624	1	3	1	1	3	4	
Lombarts Kalvill	APF1440	3	3	5	1	6	7	
London Pepping	APF0399	1	5	1	7	5	5	
Lord Grosvenor	APF1525	2	4	n.d.	5	4	5	
Lord Lambourne	APF0400	3	6	1	4	4	3	
Lord Suffield	APF1348	2	5	1	n.d.	5	5	
Ludivigs Rosenapfel	APF1258	2	3	1	1	3	3	x
Luikenapfel	APF1441	1	4	1	5	3	1	
Luisenapfel	APF0402	5	6	1	5	3	2	
Lütticher Ananaskalvill	APF1349	1	4	1	6	5	6	
Macoun	APF0405	5	7	1	7	3	5	
Maibiers Parmäne	APF0406	1	7	1	5	7	4	
Maiden Blush	APF0407	4	6	3	9	5	6	
Maigold	APF0408	4	6	1	9	4	3	
Malling Kent	APF0359	7	5	4	7	4	4	
Malvesier	APF1557	2	4	n.d.	1	2	3	
Manks Küchenapfel	APF1351	2	4	n.d.	3	5	4	
Maren Nissen	APF1442	1	7	1	4	6	5	
Margol	APF1018	3	4	1	1	2	3	
Marienwerder Gulderling	APF1443	2	7	n.d.	5	3	3	
Marina	APF1043	2	5	1	1	8	6	
Martens Sämling	APF0412	3	4	1	1	3	3	
Martha	APF1259	5	9	1	8	6	5	
Martini	APF0413	1	3	2	4	4	3	
Mauks Hybride	APF1188	2	4	1	3	3	4	
Maunzenapfel	APF0934	1	1	1	5	4	3	
Mautapfel	APF1196	2	5	n.d.	4	5	5	
McIntosh	APF0418	4	8	1	9	4	5	
McIntosh 4 n	APF0422	4	8	n.d.	n.d.	7	7	
McIntosh Giant	APF0424	4	8	n.d.	n.d.	4	6	
McIntosh Rogers	APF0426	6	7	3	9	4	5	
McIntosh Wijcik	APF0430	4	9	1	9	3	5	
Mecklenburger Königsapfel	APF1446	1	4	1	3	3	1	
Melrose	APF0433	3	5	1	7	4	5	
Meran	APF0434	7	7	8	9	6	6	
Merton Beauty	APF0435	2	4	1	4	5	4	

Cultivars	Acc. No.	Scab leaf 2012	Scab leaf 2013	Scab fruit 2012	Scab fruit 2013	Mildew leaf 2012	Mildew leaf 2013	Maximum score 3*
Merton Charm	APF0436	7	6	5	n.d.	5	6	
Metzrenette	APF1260	4	6	1	9	4	4	
Millers Seedling	APF1352	1	3	1	3	3	3	x
Milton	APF0441	2	7	1	5	5	6	
Minister von Hammerstein	APF0267	4	6	3	7	4	5	
Minjon	APF0442	2	4	1	9	5	6	
Mio	APF0443	1	1	1	3	3	3	x
Monarch	APF1354	4	3	4	5	4	3	
Monroe	APF0453	6	8	5	9	7	8	
Moringer Rosenapfel	APF1353	2	5	1	7	4	5	
Mosel-Eisenapfel	APF1261	4	5	1	1	3	1	
Multhaupts Renette	APF0445	2	5	1	7	6	6	
Murasaki	APF0454	3	5	3	7	3	4	
Muskatrenette	APF0455	1	3	1	2	5	6	
Mutsu	APF0456	7	6	5	7	7	5	
Mutterapfel	APF0457	1	4	1	5	5	4	
Nabella	APF0458	2	7	1	7	3	3	
Namfo	APF1000	6	8	7	9	7	8	
Naumburger Tiefblüte	APF1527	1	3	1	3	3	5	
Nela	APF0961	1	1	1	1	3	1	x
Nelkenapfel	APF1262	3	5	3	1	3	1	
Neuhäuser Boikenapfel	APF1447	1	7	1	5	3	3	
Neujahrsapfel	APF0463	5	8	4	n.d.	4	3	
Norda	APF0473	1	3	2	5	3	4	
Northern Spy	APF0478	4	8	n.d.	9	5	7	
Notarisapfel	APF1265	2	n.d.	1	n.d.	3	n.d.	
Obelisk	APF0481	5	7	3	9	3	5	
Oberdiecks Renette	APF0482	4	7	1	8	5	5	
Oberländer Himbeerapfel	APF1279	1	n.d.	1	n.d.	3	n.d.	
Oberlausitzer Muskatrenette	APF0254	3	1	1	3	3	3	x
Oberöstereichischer Brünnerling	APF0259	2	2	1	1	1	3	x
Ochsennase	APF1450	6	7	5	7	3	5	
Odenwälder	APF0483	2	1	2	1	3	4	
Odin	APF0485	4	5	3	9	5	7	
Oetwiler Renette	APF1528	1	2	1	1	4	4	
Ohm Paul	APF1358	1	n.d.	1	n.d.	5	n.d.	
Öhringer Blutstreifling	APF1357	3	n.d.	1	n.d.	3	n.d.	
Ontario	APF0488	5	7	1	7	8	7	
Ontario Geneva	APF0490	4	7	5	n.d.	5	7	
Orangenburg	APF0491	3	5	3	5	6	6	

Cultivars	Acc. No.	Scab leaf		Scab fruit		Mildew leaf		Maximum score 3*
		2012	2013	2012	2013	2012	2013	
Orleans Renette	APF0494	3	8	1	7	3	4	
Orthley	APF0495	9	7	9	9	7	7	
Osnabrücker Renette	APF1266	2	n.d.	1	n.d.	6	n.d.	
Otava	APF0496	3	6	2	3	5	6	
Ozark Gold	APF0500	3	7	3	9	4	6	
Pannemanns Tafelapfel	APF1019	3	6	1	3	5	7	
Parkers Pepping	APF1268	4	n.d.	1	n.d.	5	n.d.	
Peasgoods Sondergleichen	APF0502	3	5	3	n.d.	2	1	
Pfaffenhofer Schmelzling	APF1529	1	2	1	3	2	2	x
Pfirsichroter Sommerapfel	APF0506	2	1	1	3	5	7	
Pflankenapfel	APF1270	1	n.d.	1	n.d.	1	n.d.	
Pidi	APF1451	7	4	3	7	3	4	
Pigloma	APF0511	3	7	3	9	3	7	
Piglos	APF0512	5	7	3	9	3	7	
Pikant	APF0513	4	6	1	9	5	6	
Pikkolo	APF0514	4	7	1	5	5	7	
Pikora	APF0515	4	7	1	9	5	7	
Pillnitzer Roter 1	APF0766	2	5	2	5	3	3	
Pillnitzer Roter 2	APF0767	3	6	1	5	3	4	
Pimona	APF0517	3	5	1	6	6	7	
Pinett	APF0518	5	7	3	9	5	7	
Pirina	APF0521	2	6	1	5	3	4	
Pixie Crunch	APF0917	1	3	1	1	6	6	
Pohls Schlotterapfel	APF0527	1	4	1	4	5	6	
Pohorka	APF0528	2	3	n.d.	n.d.	4	7	
Pomme d'Or	APF1548	2	3	1	7	1	1	
Pommerscher Schneeapfel	APF0529	1	7	1	2	5	7	
Porzenapfel	APF1137	1	2	1	1	2	2	x
Präsident Decour	APF0888	1	1	1	5	2	1	
President Roulin	APF1087	2	4	1	3	4	5	
Prima	APF0532	1	7	1	7	1	1	
Primiera	APF1081	1	5	1	1	4	6	
Primula	APF0534	1	2	1	1	3	4	
Prinz Albrecht von Preußen	APF0007	1	2	1	1	2	3	x
Prinzenapfel	APF0536	1	3	1	3	2	3	x
Priscilla	APF0538	1	3	1	1	5	7	
Puntschapfel	APF1452	4	4	1	1	4	4	
Purpurroter Agataapfel	APF1453	1	4	1	1	2	7	
Purpurroter Cousinot	APF0541	1	1	1	1	4	3	
Purpurroter Zwiebelapfel	APF1454	1	2	1	1	3	1	x

Cultivars	Acc. No.	Scab leaf 2012	Scab leaf 2013	Scab fruit 2012	Scab fruit 2013	Mildew leaf 2012	Mildew leaf 2013	Maximum score 3*
Quinte	APF0544	2	7	1	n.d.	3	3	
Quittenförmiger Gulderling	APF1271	2	n.d.	n.d.	n.d.	4	n.d.	
Raafs Liebling	APF1564	5	6	5	5	3	5	
Rafzubin	APF0607	7	8	9	9	6	7	
Ravensberger	APF1272	2	n.d.	1	n.d.	1	n.d.	
Realka	APF0547	1	1	1	1	2	2	x
Red Boy	APF1457	2	1	1	1	5	5	
Red Delicious	APF0139	4	7	3	9	4	2	
Red Delicious Redspur	APF0140	3	7	3	n.d.	4	2	
Red Delicious Starking	APF0659	4	5	1	7	3	1	
Red Delicious Starking Starkrimson	APF0660	4	5	3	5	3	1	
Red Delicious Topred	APF0143	4	5	n.d.	n.d.	3	3	
Red Melba	APF0549	4	6	3	n.d.	3	3	
Red Topaz	APF1458	1	1	1	1	2	3	x
Red Winter	APF0554	5	5	3	3	5	5	
Reders Goldrenette	APF1360	1	n.d.	1	n.d.	2	n.d.	
Regal Prince	APF0209	6	6	6	9	6	5	
Regunde	APF0557	2	1	1	2	2	3	x
Reinette du Mans	APF0567	3	7	1	7	2	3	
Reinette Evagil	APF1089	3	6	3	4	3	4	
Reinette Franche	APF1532	2	3	n.d.	n.d.	4	5	
Reka	APF0558	1	1	1	1	3	3	x
Releta	APF0561	3	7	1	4	6	6	
Relinda	APF0562	1	1	2	2	2	3	x
Remura	APF0564	1	1	1	1	4	3	
Rene	APF0565	1	1	1	1	7	7	
Resista	APF0905	3	n.d.	1	n.d.	3	n.d.	
Retina	APF1550	1	2	1	1	3	4	
Rheingold	APF1190	3	4	1	3	3	4	
Rheinische Schafsnase	APF1405	3	n.d.	1	n.d.	4	n.d.	
Rheinischer Bohnapfel	APF0060	3	6	1	5	4	3	
Rheinischer Krummstiel	APF0944	3	6	1	3	3	4	
Rheinischer Winterrambur	APF1077	1	2	1	1	5	4	
Rheinisches Seidenhemdchen	APF1536	1	3	1	1	5	4	
Ribston Pepping	APF0577	1	3	1	1	7	7	
Riesenboiken	APF0578	1	5	1	n.d.	1	2	
Rioler Mostapfel	APF1471	3	6	1	5	4	6	
Ritters Stolz	APF0579	2	1	1	1	7	8	
Rival	APF1361	3	n.d.	3	n.d.	3	n.d.	
Rome Beauty	APF0583	5	7	3	9	5	7	

Cultivars	Acc. No.	Scab leaf 2012	Scab leaf 2013	Scab fruit 2012	Scab fruit 2013	Mildew leaf 2012	Mildew leaf 2013	Maximum score 3*
Rosemary Russet	APF1275	3	n.d.	1	n.d.	4	n.d.	
Rosenapfel von Schönbuch	APF1461	4	n.d.	1	n.d.	1	n.d.	
Rotapfel	APF1276	1	n.d.	1	n.d.	5	n.d.	
Rote Alkmene	APF0011	1	4	1	3	3	3	
Rote Alkmene Typ Rosenberg	APF1462	6	n.d.	1	n.d.	2	n.d.	
Rote Clivia	APF0589	4	7	1	9	8	7	
Rote Goldparmäne	APF0590	3	5	1	3	2	5	
Rote Sternrenette	APF0591	2	3	1	1	3	3	x
Roter Altländer Pfannkuchenapfel	APF0014	1	5	1	5	2	1	
Roter Astrachan	APF0592	3	5	1	6	2	6	
Roter Augustiner	APF1464	4	4	1	4	3	n.d.	
Roter Bellefleur	APF0593	1	3	1	1	4	3	
Roter Boskoop	APF0070	1	5	1	1	2	3	
Roter Boskoop Typ Wolf	APF1533	1	4	1	5	3	4	
Roter Cox Orange	APF0122	4	3	1	1	6	6	
Roter Eiserapfel	APF1560	2	6	n.d.	1	3	1	
Roter Finkenwerder	APF1534	1	4	1	3	3	1	
Roter Fuchs	APF1362	2	n.d.	1	n.d.	2	n.d.	
Roter Gravensteiner	APF0117	2	6	1	n.d.	4	5	
Roter Hauptmann	APF0595	4	7	3	9	3	5	
Roter Helios	APF0273	5	7	4	6	4	4	
Roter Holsteiner Cox	APF1427	1	3	1	3	6	5	
Roter Jakob Lebel	APF1430	4	5	1	1	3	4	
Roter Lord Lambourne	APF0600	3	6	1	1	3	4	
Roter Metternich	APF1280	2	n.d.	1	n.d.	3	n.d.	
Roter Mond	APF1466	3	7	1	9	2	3	
Roter Oldenburg	APF0487	7	7	7	9	3	4	
Roter Säfstaholms	APF0601	1	2	1	n.d.	4	4	
Roter Sossenheimer	APF1287	3	4	1	3	1	2	
Roter Stettiner	APF0727	2	6	1	3	2	3	
Roter Trierer Weinapfel	APF1363	2	n.d.	1	n.d.	3	n.d.	
Roter Winterkalvill	APF1136	9	8	7	9	4	6	
Roter Ziegler	APF1535	1	3	1	n.d.	3	2	
Rotfranch	APF1364	1	n.d.	1	n.d.	3	n.d.	
Rotgestreifte Gelbe Schafsnase	APF1048	1	3	1	5	5	6	
Röthaer Blenheim	APF0059	4	7	3	9	3	3	
Rubin	APF0606	6	7	5	n.d.	3	5	
Rubinstar	APF1135	7	7	6	9	7	7	
Ruhm aus Kelsterbach	APF1281	1	4	1	4	3	3	
Ruhm aus Kirchwerder	APF1365	1	1	1	3	4	3	

Cultivars	Acc. No.	Scab leaf 2012	Scab leaf 2013	Scab fruit 2012	Scab fruit 2013	Mildew leaf 2012	Mildew leaf 2013	Maximum score 3*
Sächsischer Königsapfel	APF0370	2	6	1	3	4	6	
Sacramentsappel	APF1367	5	4	1	n.d.	5	4	
Safranapfel	APF0609	3	6	1	7	5	5	
Salemer Klosterapfel	APF1469	1	4	1	1	3	3	
Salome	APF0611	1	3	1	5	6	5	
Sämling von Hain	APF0613	3	4	1	9	3	4	
Sansa	APF0927	1	3	1	2	6	7	
Santana	APF1052	1	3	1	3	5	5	
Sauergrauech	APF1470	1	3	3	3	3	4	
Schieblers Taubenapfel	APF1472	1	5	1	7	5	4	
Schlesischer Rotborsdorfer	APF0617	2	6	1	7	3	5	
Schlotterapfel	APF1473	2	5	1	5	3	3	
Schmalzprinz	APF0618	1	3	2	1	3	3	x
Schmidtbergers Renette	APF1283	4	4	1	3	4	5	
Schneica	APF0332	5	7	4	9	7	8	
Schneiderapfel	APF0945	1	2	n.d.	3	3	4	
Schöner aus Bath	APF0619	3	3	1	3	3	3	x
Schöner aus Berwangen	APF1539	1	3	1	1	3	4	
Schöner aus Burscheid	APF1540	1	3	1	1	4	3	
Schöner aus Elmpt	APF1538	1	1	1	1	2	1	x
Schöner aus Hadeln	APF1541	1	3	1	1	3	3	x
Schöner aus Haseldorf	APF0620	1	4	n.d.	2	4	5	
Schöner aus Herrnhut	APF0275	1	4	1	1	4	5	
Schöner aus Lutten	APF1475	6	6	1	3	5	6	
Schöner aus Miltenberg	APF0265	2	5	1	n.d.	3	3	
Schöner aus Nordhausen	APF0474	2	4	1	9	3	3	
Schöner aus Pontoise	APF0623	1	4	n.d.	4	4	4	
Schöner aus Wiedenbrück	APF1369	1	3	1	5	4	3	
Schöner aus Wiltshire	APF1117	1	1	1	1	2	3	x
Schöner von Mlejew	APF0621	1	5	1	5	3	3	
Schwarzschillernder Kohlapfel	APF1465	1	7	1	3	2	n.d.	
Schweizer Orangenapfel	APF0625	2	4	1	n.d.	4	5	
Seestermüher Zitronenapfel	APF0627	3	3	3	3	4	4	
Shampion	APF0631	9	7	4	9	3	5	
Shin Indo	APF0632	4	6	2	6	4	5	
Shinsei	APF0633	8	7	8	9	3	4	
Siebenschläfer	APF1370	2	3	1	3	3	3	x
Signe Tillisch	APF0635	4	7	2	9	5	5	
Simirenko	APF0469	6	7	4	9	7	7	
Slava Pobeditelâam	APF0639	1	3	2	4	4	3	

Cultivars	Acc. No.	Scab leaf 2012	Scab leaf 2013	Scab fruit 2012	Scab fruit 2013	Mildew leaf 2012	Mildew leaf 2013	Maximum score 3*
Sohlander Streifling	APF0641	1	3	1	3	4	3	
Sommermaschanzker	APF1479	2	5	1	1	3	4	
Sommerparmäne	APF1368	3	7	3	9	4	7	
Sommerregent	APF1192	3	7	2	9	2	4	
Sonnenwirtsapfel	APF1371	1	3	1	1	1	1	x
Southern Snap	APF1122	5	8	3	9	5	4	
Spartan	APF0644	3	6	1	n.d.	3	3	
Spätblühender aus Bockedra	APF1481	2	3	1	4	3	3	
Spätblühender Taffetapfel	APF1372	3	1	1	3	3	3	x
Spencer	APF0652	3	8	1	9	5	7	
Sperenza	APF1034	3	8	1	9	5	6	
Spielberger Wieslesapfel	APF1542	2	5	1	1	2	3	
Stahls Winterprinz	APF0655	1	6	1	3	3	4	
Stayman Winesap Blackstayman	APF0663	5	7	4	9	7	6	
Stedinger Prinz	APF1482	4	4	1	5	1	3	
Steinbacher	APF1288	1	1	1	1	6	6	
Steirischer Maschanzker	APF1049	3	5	2	5	4	3	
Stenbock	APF0665	1	4	1	5	3	4	
Stern von Bühren	APF0956	1	3	n.d.	5	3	6	
Stina Lohmann	APF0075	2	3	1	7	1	3	
Strauwalds Parmäne	APF0666	1	7	1	1	3	4	
Süderhex	APF1375	1	3	1	2	3	2	x
Sulinger Grünling	APF1543	2	5	1	1	1	2	
Summerland	APF0667	3	7	1	7	5	7	
Summerred	APF0668	7	7	2	n.d.	4	5	
Sunrise	APF1133	5	6	6	n.d.	3	5	
Suntan	APF0670	3	5	1	n.d.	5	6	
Süßapfel	APF0895	1	3	1	3	3	4	
Tannenkrüger	APF1545	4	5	1	6	2	4	
Telamon	APF0677	6	9	5	9	6	1	
Tenroy	APF0605	6	7	4	9	5	6	
Thurgauer Weinapfel	APF1376	3	4	1	1	3	3	
Topaz	APF0963	1	1	1	1	4	5	
Trajan	APF0682	6	9	6	9	6	3	
Transparent aus Croncels	APF0126	1	5	1	9	3	5	
Trendelburger Kalvill	APF1290	3	3	1	4	5	7	
Trenklesämling	APF1488	2	6	1	5	3	1	
Trennfurter Renette	APF1558	2	3	n.d.	1	6	5	
Triumph aus Luxemburg	APF1291	2	3	1	3	4	3	
Tsugaru	APF0929	3	4	2	9	6	4	

Cultivars	Acc. No.	Scab leaf 2012	Scab leaf 2013	Scab fruit 2012	Scab fruit 2013	Mildew leaf 2012	Mildew leaf 2013	Maximum score 3*
Tulpenapfel	APF1489	3	5	n.d.	3	3	3	
Tuscan	APF0687	6	9	5	9	6	3	
Tydemans Oktober-Pepping	APF0688	3	4	1	4	5	4	
Uelzener Kalvill	APF1292	7	7	4	8	3	4	
Uelzener Rambur	APF1293	3	5	1	1	3	1	
Uhlhorns Augustkalvill	APF1377	1	n.d.	1	n.d.	4	n.d.	
Ulmer Polizeiapfel	APF1491	1	3	1	8	3	2	
Undine	APF0693	4	7	3	3	6	6	
Unseldapfel	APF1492	1	3	n.d.	4	4	3	
Uphuser Titjenapfel	APF1294	2	n.d.	1	n.d.	2	n.d.	
Vaterapfel	APF1295	2	n.d.	1	n.d.	2	n.d.	
Viking	APF0698	4	8	1	9	3	3	
Virginia Crab	APF1024	3	8	1	9	1	1	
Wachendorfer Renette	APF1546	1	5	1	1	4	4	
Wagenerapfel	APF0704	6	5	6	n.d.	8	7	
Warner's King	APF1326	2	6	1	5	7	7	
Wealthy 4 n Loop	APF0712	3	4	1	n.d.	4	3	
Weidners Goldrenette	APF1378	2	n.d.	1	n.d.	3	n.d.	
Weißer Astrachan	APF1199	2	3	1	n.d.	3	4	
Weißer Matapfel	APF1509	2	n.d.	n.d.	n.d.	2	3	
Weißer Winterglockenapfel	APF0235	1	5	1	5	1	3	
Weißer Winterkalvill	APF0715	8	7	9	9	6	6	
Weißer Wintertaffetapfel	APF0716	4	6	3	7	5	3	
Weißkante	APF1549	1	4	1	1	2	3	
Welschisner	APF0718	1	1	1	1	3	2	x
Welschweinling	APF0719	1	3	n.d.	n.d.	2	1	
Werdersche Wachsrenette	APF1495	4	6	3	9	3	3	
Westfälische Tiefblüte	APF1298	2	n.d.	1	n.d.	1	n.d.	
Westfälischer Gulderling	APF0958	1	3	1	1	2	3	x
Wettringer Taubenapfel	APF1299	1	n.d.	1	n.d.	3	n.d.	
Wiesenapfel	APF1300	1	n.d.	1	n.d.	5	n.d.	
Wildeshausener Goldrenette	APF0959	1	4	1	3	1	1	
Wilkenburger Herbstrenette	APF1380	2	n.d.	1	n.d.	5	n.d.	
Winesap	APF0700	6	7	9	9	3	5	
Winston	APF0722	4	6	1	5	4	4	
Winterbananenapfel	APF0723	7	6	6	n.d.	5	4	
Wintergewürzapfel	APF1496	1	5	1	6	4	5	
Wöbers Rambur	APF0728	3	6	1	9	3	3	
Wohlschmecker aus Vierlanden	APF1381	2	n.d.	1	n.d.	3	n.d.	
Worcester Parmäne	APF0730	1	5	1	5	4	4	

Cultivars	Acc. No.	Scab leaf		Scab fruit		Mildew leaf		Maximum score 3*
		2012	2013	2012	2013	2012	2013	
Wrixparent	APF0732	7	3	1	n.d.	7	5	
York Imperial Colora Red York	APF0113	2	5	1	3	4	3	
Zabergäurenette	APF0736	2	6	1	1	5	5	
Zigeunerin	APF1499	4	n.d.	5	3	2	3	
Zimtrenette	APF0738	2	5	1	1	5	6	
Zuccalmaglios Renette	APF0740	3	5	1	3	5	5	

n.d. not determined

* all data available

Table S2: List of molecular markers used for genotyping. The respective fluorescent dye, the composition of multiplexes and source of primer sequences are shown.

Marker	Dye	Mulitiplex (MP)	Reference
AT20Scar	6-FAM	MP1/Res_mild	Dunemann, Peil, Urbanietz and Garcia-Libreros [27]
CH02b07	6-FAM	MP4	https://sites.unimi.it/camelot/hidras/
CH02b10	ATTO 550	MP5	https://sites.unimi.it/camelot/hidras/
CH02c02a	ATTO 565	MP1	https://sites.unimi.it/camelot/hidras/
CH02c06	ATTO 565	MP5	https://sites.unimi.it/camelot/hidras/
CH02d12	ATTO 532	MP6/Res_mild	https://sites.unimi.it/camelot/hidras/
CH02f06	ATTO 550	MP3	https://sites.unimi.it/camelot/hidras/
CH03c02	6-FAM	MP1/Res_mild	https://sites.unimi.it/camelot/hidras/
CH03d01	ATTO 565	MP2	https://sites.unimi.it/camelot/hidras/
CH04f03	ATTO 532	MP5	https://sites.unimi.it/camelot/hidras/
CH05e03	6-FAM	MP5	https://sites.unimi.it/camelot/hidras/
CH04h02	ATTO 550	Res_mild	https://sites.unimi.it/camelot/hidras/
CH-Vf1	ATTO 532	MP4	Vinatzer*, et al.* [52]
FMACH_Vm2	6-FAM	MP3	Cova, Bandara, Liang, Tartarini, Patocchi, Troggio, Velasco and Komjanc [26]
FMACH_VM3	ATTO 532	MP2	Cova, Bandara, Liang, Tartarini, Patocchi, Troggio, Velasco and Komjanc [26]
HB09	ATTO 550	MP2	Soufflet-Freslon, Gianfranceschi, Patocchi and Durel [28]
Hi07h02	ATTO 532	MP1	https://sites.unimi.it/camelot/hidras/
Hi08e04	ATTO 565	MP6	https://sites.unimi.it/camelot/hidras/
NH030a	ATTO 550	MP4	Yamamoto*, et al.* [53]
NZmsCN943818	ATTO 565	MP3	https://sites.unimi.it/camelot/hidras/
OPB18SCAR	ATTO 532	MP5	Bus, Laurens, van de Weg, Rusholme, Rikkerink, Gardiner, Bassett, Kodde and Plummer [20]
OPL19SCAR	ATTO 532	MP2	Bus, Laurens, van de Weg, Rusholme, Rikkerink, Gardiner, Bassett, Kodde and Plummer [20]
Pl2_F1/R1	6-FAM	MP1/Res_mild	this study
Rvi18-SSR	ATTO 550	MP1	Soriano*, et al.* [54]
SSR-23.03	6-FAM	MP1	Padmarasu, Sargent, Jaensch, Kellerhals, Tartarini, Velasco, Troggio and Patocchi [25]
SSR-23.17	ATTO 532	MP3	Padmarasu, Sargent, Jaensch, Kellerhals, Tartarini, Velasco, Troggio and Patocchi [25]

SSR-24.91	6-FAM	MP2	Padmarasu, Sargent, Jaensch, Kellerhals, Tartarini, Velasco, Troggio and Patocchi [25]
T6	ATTO 565	MP3	Gygax, Gianfranceschi, Liebhard, Kellerhals, Gessler and Patocchi [21]
Vg12_SSR	6-FAM	MP6	Cova*, et al.* [55]
Vg15_SSR	ATTO 550	MP6	Cova, Lasserre-Zuber, Piazza, Cestaro, Velasco, Durel and Malnoy [55]
Vr2C_UTR	ATTO 565	MP2	this study
